# Recent Advances in the Diagnosis and Management of Clinically Significant Portal Hypertension in Liver Cirrhosis: A Clinical Review

**DOI:** 10.3390/biomedicines14051133

**Published:** 2026-05-16

**Authors:** Jan Christoph Schumacher, Joshua Leutiger, Anna Martin, Münevver Demir, Christoph Neumann-Haefelin, Philipp Kasper

**Affiliations:** 1 Department of Gastroenterology and Hepatology, Faculty of Medicine and University Hospital Cologne, University of Cologne, 50937 Cologne, Germany; jan.schumacher@uk-koeln.de (J.C.S.); joshua.leutiger@uk-koeln.de (J.L.); anna.martin@uk-koeln.de (A.M.); christoph.neumann-haefelin@uk-koeln.de (C.N.-H.); 2 Department of Hepatology and Gastroenterology, Campus Virchow Clinic and Campus Charité Mitte, Charité University Medicine Berlin, 13353 Berlin, Germany; muenevver.demir@charite.de

**Keywords:** liver cirrhosis, hepatic decompensation, portal hypertension, carvedilol, non-selective beta-blockers, non-invasive testing

## Abstract

Clinically significant portal hypertension (CSPH) is a key driver of decompensation events and complications in patients with liver cirrhosis. The manifestation of hepatic decompensation is, in turn, associated with a drastic deterioration in prognosis in this vulnerable population. Therefore, a timely identification of patients at risk as well as an optimal drug treatment are key elements in the therapeutic management of patients with cirrhosis to improve survival. While invasive measurements represent the gold standard for assessing portal hypertension, significant progress has been made in recent years in developing non-invasive methods for evaluating CSPH, including the NICER and the ANTICIPATE model. These models, combining variables such as liver and spleen stiffness in combination with biomarkers such as platelet count for risk prediction, reliably identify patients with CSPH who are at risk for hepatic decompensation. In addition to advances in diagnostics, new evidence has emerged regarding optimal drug management strategies. Non-selective beta blockers are an important treatment option, even though a certain proportion of patients respond inadequately. Therefore, updated strategies are needed to identify these non-responders and improve treatment effectiveness. This review article provides a comprehensive overview of modern diagnostic approaches for CSPH, describes current management strategies including updated approaches, and provides an outlook on emerging potential treatment options.

## 1. Definition of Portal Hypertension

Liver cirrhosis represents the terminal stage of chronic liver disease and can be clinically divided into a compensated and a decompensated stage, which significantly differs in the incidence of complications and overall survival [1,2]. Decompensated cirrhosis becomes clinically apparent through the manifestation of ascites, hepatic encephalopathy, jaundice or portal hypertensive hemorrhage (e.g., esophageal variceal bleeding). While the median survival of patients with compensated cirrhosis and optimal therapy is usually over 10 years, the occurrence of a decompensating (e.g., ascites) event is associated with a significant deterioration in prognosis [2,3,4,5].

A central driving factor in the pathogenesis of hepatic decompensation is the extent of underlying portal hypertension [1]. Therefore, early identification and adequate management of clinically significant portal hypertension (CSPH) is of great clinical and prognostic relevance.

Portal vein pressure is expressed as the pressure gradient between the portal vein and the inferior vena cava, which is referred to as hepatic venous pressure gradient (HVPG).

Portal hypertension is defined as a pathological increase in HVPG > 5 mmHg [6]. A HVPG ≥ 10 mmHg has been identified as the strongest predictor for the development of hepatic decompensation and esophageal varices and defines the presence of CSPH [3].

According to the anatomical location of the blood flow obstruction and the site of increased vascular resistance, portal hypertension can be classified into pre-, intra- and post-hepatic portal hypertension. Intrahepatic causes can further be subcategorized as presinusoidal, sinusoidal, and postsinusoidal portal hypertension.

When considering the causes of portal hypertension, these vary geographically and include advanced liver diseases, as well as infectious (e.g., Schistosomiasis), vascular, and metabolic diseases.

In Western nations, liver cirrhosis is the predominant cause of portal hypertension, followed by portal vein thrombosis [7].

This review aims to provide a comprehensive and clinically focused overview of current diagnostic and therapeutic strategies for portal hypertension in patients with cirrhosis, integrating recent advances in non-invasive diagnostics into an up-to-date algorithm for everyday clinical practice. Furthermore, emerging diagnostic approaches, including artificial intelligence-based applications, radiomics and endo-hepatology are discussed alongside future therapeutic concepts in the context of personalized risk stratification and management.

## 2. Materials and Methods

A structured literature search was conducted in PubMed and Google Scholar up to 6 April 2026. Relevant studies were identified by use of a combination of the following keywords and MeSH-terms: liver cirrhosis, portal hypertension, non-invasive testing, hepatic decompensation. Additional references were identified through citation screening and expert knowledge. Only full-text articles and abstracts in English were considered.

## 3. Pathogenesis

**Figure 1:**
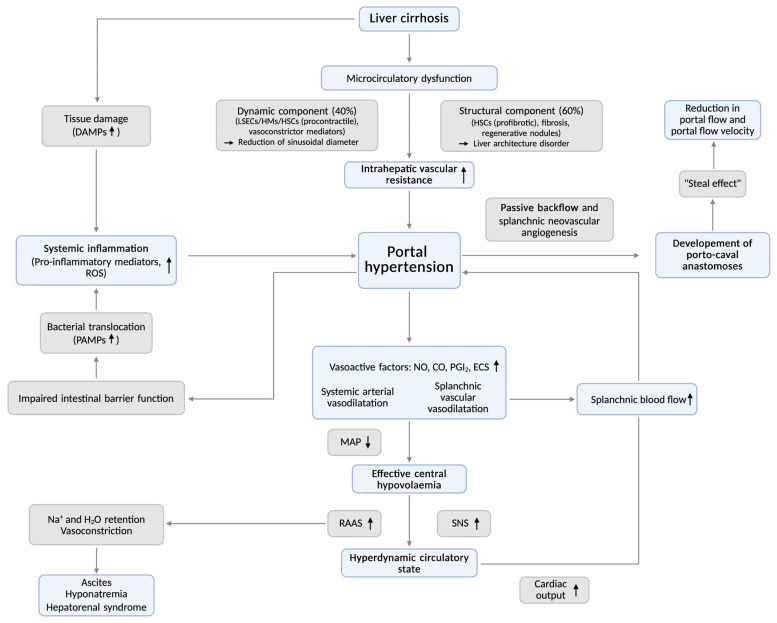
Pathogenesis of portal hypertension. Portal hypertension results from increased intrahepatic vascular resistance due to structural and dynamic components, in combination with augmented portal venous inflow driven by splanchnic vasodilation and a hyperdynamic circulatory state. These hemodynamic alterations are closely linked to systemic inflammation, which further exacerbates portal hypertension and contributes to clinical decompensation. Grey arrows indicate the main pathophysiological pathways contributing to the development of portal hypertension. Black arrows indicate an increase or decrease in specific pathophysiological aspects or mechanisms. The differently colored boxes are used to visually distinguish distinct pathophysiological mechanisms (grey boxes) from clinical consequences (blue boxes). Abbreviations: CO, Carbon monoxide; DAMPs, Damage-associated molecular patterns; ECS, Endocannabinoid system; HMs, Hepatic macrophages; HSC, Hepatic stellate cells; H_2_O, water; LSECs, Liver sinusoidal endothelial cells; MAP, Mean arterial pressure; Na^+^, Sodium; NO, Nitric oxide; PAMPs, Pathogen-associated molecular patterns; PGI_2_, Prostacyclin (Prostaglandin I_2_); RAAS, Renin–angiotensin–aldosterone system; ROS, Reactive oxygen species; SNS, Sympathetic nervous system. Created in BioRender. Kasper, P. (2026) https://BioRender.com/43hnfgo, (accessed on 31 March 2026).

In patients with liver cirrhosis, portal hypertension develops primarily as a result of increased intrahepatic vascular resistance (IHVR) (Figure 1). The increase in IHVR is driven by two principal mechanisms. First, there is a structural distortion of the liver architecture, caused by fibrotic remodeling due to liver damage. Secondly, there is intrahepatic vasoconstriction, which is caused by dysfunction of sinusoidal endothelial cells, a contraction of myofibroblasts and an excess of vasoconstricting mediators with a simultaneous deficiency of vasodilating mediators (e.g., nitric oxide (NO)). These alterations result in passive congestion within the portal venous system and the splanchnic area, the so-called ‘backward flow’ component, which leads to the compensatory formation or opening of porto-caval anastomoses between the portal venous and systemic circulation. Concurrently, excessive release of vasoactive substances (e.g., NO) induces splanchnic and systemic vasodilation [8,9].

The aforementioned changes lead to sequestration of blood within the portal-mesenteric circulation, thereby reducing the effective arterial blood volume and resulting in central hypovolemia. This reduces cardiac preload and stroke volume, triggering a compensatory response, characterized by tachycardia (increasing cardiac output), a reactive increase in sympathetic tone and activation of the renin–aldosterone–angiotensin system (RAAS). This ultimately leads to a hyperdynamic circulatory state and increased splanchnic blood flow, the so-called ‘in-flow’ or ‘forward-flow’ component [8,9,10,11].

In the presence of multiple and large porto-caval anastomoses, a ‘steal effect’ can be observed, leading to a reduction in portal blood flow and portal flow velocity [12,13].

Hemodynamic changes are accompanied by a systemic inflammatory response, which plays a critical role in the development of decompensation events and associated organ dysfunction [8]. In liver cirrhosis, this systemic inflammatory reaction is attributed, on the one hand, to direct liver cell damage caused by various damaging factors (e.g., alcohol or viral infections) with consecutive release of damage-associated molecular patterns (DAMPs), and, on the other hand, to abnormal translocation of bacteria and microbial components known as pathogen-associated molecular patterns (PAMPs) or microbiome-associated molecular patterns (MAMPs) from the intestinal lumen into the portal circulation [10,11]. Within the liver, PAMPs and DAMPs bind to specific receptor systems (pathogen recognition receptors), such as Toll-Like Receptors (TLR), and stimulate immune cells. This activation is associated with an increased release of pro-inflammatory mediators (e.g., pro-inflammatory cytokines), activation of inflammatory signaling pathways, and the recruitment of further immune cells, all of which ultimately maintain and intensify the prevailing underlying inflammatory response. The inflammatory reaction is accompanied by an increased release of reactive oxygen species (ROS) and an imbalance of vasoconstrictive and vasodilatory mediators, which together exacerbate intrahepatic resistance.

Recent clinical studies have demonstrated that the degree of systemic inflammation correlates with the extent of portal hypertension and constitutes a major driver of clinical deterioration and decompensating events [14].

## 4. Diagnostic Approaches

### 4.1. Invasive Assessment

HVPG measurement is considered the gold standard for diagnosing CSPH [3]. The HVPG is measured in a minimally invasive procedure, whereby a balloon catheter is placed transjugularly into the hepatic vein. In this way, the difference between the wedged hepatic venous pressure (WHVP) and the free hepatic venous pressure (FHVP), which represent the portal and central venous pressures, respectively, is measured.

HVPG measurement is the most frequently used and best validated method for estimating portal vein pressure and assessing the risk of developing hepatic complications. While gastro-esophageal varices typically develop from an HVPG ≥ 10 mmHg, ascites or portal hypertensive bleeding usually manifest from an HVPG ≥ 12 mmHg. An HVPG ≥ 16 mmHg is associated with a substantially increased liver-related mortality, and an HVPG ≥ 20 mmHg markedly elevates the risk of re-bleeding in patients with acute variceal hemorrhage [15].

In addition, an HVPG ≥ 10 provides important prognostic information, as it can predict the risk of short-term hepatic decompensation in patients with compensated cirrhosis [1,16].

A reduction of the HVPG to <12 mmHg or by ≥10–20% from baseline significantly decreases the risk of variceal bleeding and other portal hypertensive complications [15,17].

When performing an HVPG measurement, it should be considered that it primarily provides consistent results in the setting of sinusoidal portal hypertension. The presence of other components of portal hypertension (e.g., pre- or post-sinusoidal) can lead to a normal HVPG gradient despite the presence of CSPH, which should be taken into account when interpreting the results.

A subgroup requiring special consideration, when interpreting the results are patients with metabolic dysfunction-associated steatotic liver disease (MASLD). In these patients clinically relevant decompensations may already be observed at HVPG values < 10 mmHg [18,19]. Recent studies have shown that HVPG measurement tends to underestimate the portal pressure in MASLD, likely due to a substantial pathogenic pre-sinusoidal component (in addition to the sinusoidal component) in these patients. Factors contributing to the complex changes in portal hemodynamics in MASLD include hepatic sinusoidal narrowing due to hepatocellular lipid accumulation and ballooning, sinusoidal endothelial cell dysfunction, periportal fibrosis, inter-sinusoidal shunts, increased sinusoidal capillarization, microthrombosis, and a presinusoidal contraction [20,21]. However, further studies are needed to gain a better understanding of the underlying pathomechanisms and to develop new methods which can adequately evaluate the degree of portal hypertension in patients with MASLD [20,21,22,23].

### 4.2. Non-Invasive Assessment of CSPH

Since invasive methods for detecting portal hypertension require a high degree of technical expertise and special equipment, they are often only available at specialized centers.

Consequently, the identification of CSPH through widely accessible, reliable, and cost-effective non-invasive tests (NITs) has become increasingly important. A variety of NITs—broadly classified into serum biomarkers and elastography-based approaches—have been developed and extensively investigated. The Baveno consensus criteria, which are regularly developed by experts in the field and are currently reflected in the Baveno VII criteria, provide an overview of suitable non-invasive tests and propose criteria for the non-invasive diagnosis of CSPH [3].

The aim of non-invasive diagnostics is the early detection of patients with CSPH, who are at high risk of hepatic decompensation requiring close monitoring.

### 4.3. Liver Stiffness Measurement and Platelet Count

Elastography-based techniques rely on the mechanical and elastic properties of tissues following deformation or displacement. A defined mechanical or acoustic stimulus induces microdisplacements within the tissue, generating shear waves. Their propagation velocity can be measured by ultrasound and increases with tissue stiffness. While healthy liver tissue is highly compliant, fibrotic liver tissue exhibits an increased stiffness. Therefore liver stiffness, assessed by elastography, reflects the extent of hepatic fibrosis and correlates with portal pressure [24,25]. However, liver stiffness is not solely determined by fibrosis but may also be affected by other pathological or physiological processes within the liver. Potential confounding factors, including local or systemic inflammation (e.g., acute viral hepatitis), obstructive cholestasis, hepatic venous congestion, infiltrative liver disease and postprandial increases in portal venous perfusion, should therefore be considered when interpreting the results [24,25,26]. In the following, this review focuses on liver stiffness measurement (LSM) by vibration-controlled transient elastography (TE), as it currently represents the most extensively validated method [24].

The ability of LSM to identify CSPH is supported by a strong body of evidence. A recent meta-analysis, including 26 studies and 4337 patients who underwent both LSM by TE and HVPG measurement, showed a good correlation between LSM and HVPG (R = 0.70; range 0.36–0.86), with an area under the hierarchical summary receiver operating characteristic (HSROC) curve of 0.91 (95% CI 0.88–0.93) for the diagnosis of CSPH by LSM [27].

The Baveno VII criteria propose a ‘rule of five’, whereby LSM values of 5, 10, 20 and 25 kPa are associated with a progressively higher relative risk of hepatic decompensation and liver-related death [3]. A combination of LSM and platelet count, as an indirect marker of portal hypertension, has been demonstrated to be highly accurate in the assessment of CSPH [3,6].

**Figure 2:**
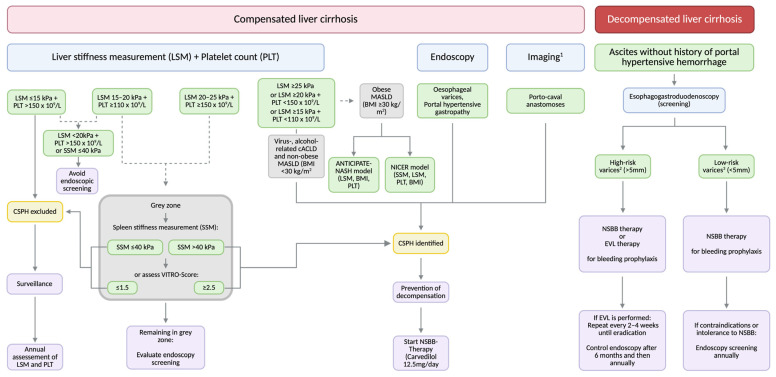
Diagnostic and treatment algorithm for patients with or without clinically significant portal hypertension (CSPH). ^1^ Abdominal ultrasound or computed tomography. ^2^ High-risk varices: varices > 5 mm, evidence of red color signs, Child–Pugh class C. ^3^ Low-risk varices: varices < 5 mm, no evidence of red color signs, no Child–Pugh class C. Solid arrows indicate the main diagnostic or therapeutic pathway, whereas dashed arrows indicate alternative or additional assessment steps. The differently colored boxes distinguish the main categories shown in the algorithm: disease stage (red), diagnostic procedures or tools (blue), diagnostic criteria or findings (green), additional assessment steps (grey), clinical decision points (yellow), management recommendations (purple). Abbreviations: BMI, Body mass index; cACLD, compensated advanced chronic liver disease; CSPH, Clinically significant portal hypertension; EVL, Endoscopic variceal ligation; kPa, Kilopascal; LSM, Liver stiffness measurement; MASLD, Metabolic dysfunction-associated steatotic liver disease; NASH, Non-alcoholic statohepatitis; NICER, Non-invasive CSPH estimated risk (model); NSBB, Non-selective beta-blocker; PLT, platelet count; SSM, Spleen stiffness measurement; VITRO, Von Willebrand Factor Antigen/Thrombocyte Ratio. Created in BioRender. Kasper, P. (2026) https://BioRender.com/qzhytmw (accessed on 31 March 2026).

According to the Baveno VII consensus criteria, CSPH can be ‘ruled in’ in patients with a LSM value ≥ 25 kPa (positive predictive value > 90%) or ‘ruled out’ in patients with a LSM ≤ 15 kPa together with a platelet count ≥ 150 × 10^9^/L (negative predictive value > 90%) (Figure 2) with high certainty (21).

Furthermore, CSPH should be presumed in patients with a LSM value between 20 and 25 kPa in combination with a platelet count < 150 × 10^9^/L or a LSM value between 15 and 20 kPa along with a platelet count < 110 × 10^9^/L [6].

The Baveno VII criteria have been validated in multiple clinical cohorts of patients with compensated advanced chronic liver disease (cACLD). In these studies, the Baveno VII ‘rule out’ criteria showed a high negative predictive value for hepatic decompensation, whereas the risk of decompensation was significantly higher in patients fulfilling the ‘rule in’ criteria [28,29,30]. In a large retrospective cohort of 17,076 patients with chronic liver disease without prior decompensation or hepatocellular carcinoma, Vutien et al. further demonstrated that the ‘rule of five’ categories were strongly associated with a higher risk of hepatic decompensation (HR: 1.77, 95% CI: 1.68–1.87) and death (HR: 1.30, 95% CI: 1.25–1.35) among patients with suspected CSPH according to the Baveno VII criteria [31]. Vutien and coworkers additionally showed, that the subgroup of patients with LSM of 50–75 kPa (‘critical’ CSPH) has approximately double the risk of death and hepatic decompensation than patients with a LSM between 25 and 49.9 kPa [31].

However, it has to be noted that these reference values only apply to patients with advanced liver disease caused by viral hepatitis or alcohol, and non-obese patients (body mass index (BMI) < 30 kg/m^2^) with metabolic dysfunction-associated steatotic liver disease (MASLD).

In obese patients with cirrhosis due to MASLD, LSM tends to overestimate the presence of CSPH [32]. Thus, the ANTICIPATE-NASH model, incorporating LSM, platelet count, and BMI has been established to refine CSPH risk estimation in this distinctive subgroup [3,6]. In a recent meta-analysis, Bañares et al. showed that applying a CSPH risk threshold of ≥75% from the ANTICIPATE-NASH model significantly increased the PPV for CSPH in obese patients with MASLD from 0.67 to 0.83 (*p* < 0.001), compared to a CSPH ‘rule-in’ strategy based on LSM ≥ 25 kPa alone, thereby significantly enhancing the diagnostic accuracy of non-invasive CSPH risk assessment in this patient population [33].

Overall, the combination of LSM and platelet count represents a non-invasive, cost-effective and easily repeatable method for diagnosing CSPH. Although the availability of TE varies across regions, LSM requires substantially less technical expertise and fewer resources than HVPG measurement. However, potential confounders (hepatic inflammation, hepatic congestion, etc.) should be considered when interpreting the results (Table 1).

Notably, while the aforementioned cut-off values for the exclusion or confirmation of CSPH are highly specific, a significant proportion of patients (approx. 50%) remain unclassified, leaving them in a state of diagnostic uncertainty and diagnostic ‘grey zone’ [34,35].

### 4.4. Spleen Stiffness Measurement

Spleen stiffness measurement (SSM) has emerged as a valuable additional surrogate parameter of PH. Increased portal pressure impairs splenic venous outflow, leading to splenic congestion and fibrotic remodeling. In contrast to LSM, which primarily reflects liver fibrosis as an estimate of static intrahepatic resistance to the portal blood flow, SSM may more closely mirror the hemodynamic consequences of PH, including the dynamic (pre-)sinusoidal vasoconstriction as a component of the splenic outflow obstruction, as well as the PH-induced splenic fibrosis and increased splanchnic inflow related to the hyperdynamic circulation [26,36].

The addition of spleen stiffness measurement (SSM) to the established Baveno criteria (LSM value and platelet count) can substantially reduce the diagnostic ‘grey zone’. According to the recent Baveno VII consensus recommendations, an SSM value of <21 kPa can rule out the presence of CSPH, whereas a value of >50 kPa indicates the presence of CSPH with a high degree of probability [3].

More recently, Dajti et al. demonstrated that it was possible to reduce the ‘grey zone’ to 7–15% by using an extended calculation model consisting of LSM, platelet count, and SSM [34]. In this approach, CSPH was excluded when at least two of the following criteria were met: LSM value ≤ 15 kPa, platelet count ≥ 150 × 10^9^/L, and SSM value ≤ 40 kPa. Conversely, CSPH was confirmed when at least two of the following criteria were fulfilled: LSM value > 25 kPa, platelet count < 150 × 10^9^/L, and SSM value > 40 kPa [37].

The recently introduced non-invasive CSPH estimated risk model (NICER model) integrates SSM, LSM, platelet count, and BMI into a single prediction model. Across different etiologies of chronic liver disease, including obese and non-obese MASLD, alcoholic liver disease and viral hepatitis, the NICER model demonstrated significantly superior discriminative ability compared with the Baveno VII criteria and the ANTICIPATE-NASH model, highlighting its particular value in clinical practice [35].

Recent studies have demonstrated that non-invasive prognostic models (NICER model, ANTICIPATE-NASH and the Baveno VII criteria) exhibit predictive performance comparable to HVPG measurements in estimating the risk of hepatic decompensation [16,38]. In a large multicenter cross-sectional cohort of 358 patients with Child–Pugh A cirrhosis, the NICER model and ANTICIPATE±NASH models demonstrated C-indices and area under the receiver operating characteristics curves (AUROCs) similar to those of invasive HVPG measurement for predicting the risk of hepatic decompensation within 1 year. Their predictive accuracy improved further upon incorporating serum albumin, as a biochemical marker for hepatic synthetic function, enabling discrimination between patients at negligible risk and those with an approximate 15% 1-year risk of decompensation [38].

### 4.5. Blood Based Tests

Another non-invasive tool for diagnosing CSPH is the VITRO score, calculated by dividing the von Willebrand factor antigen (vWF-Ag, expressed in %), a marker for endothelial dysfunction, by the platelet count. Similar to other non-invasive markers, the VITRO score correlates with the prevalence of CSPH and additionally yields prognostic information regarding the risk of decompensation and mortality in patients with liver cirrhosis [39,40].

The sequential application of Baveno VII criteria and VITRO Score significantly improved the diagnostic accuracy of CSPH, and reduced the number of ‘unclassifiable’ patients within the diagnostic ‘grey zone’ to <15%, thereby enhancing risk stratification and refining prognostication [41].

However, because vWF is an acute-phase protein and subject to diurnal variation, its levels may be influenced by inflammation as well as endothelial dysfunction. Therefore, further studies to determine its suitability as a valid biomarker are needed [42,43].

Recently it has been shown that based on routine laboratory parameters, machine-learning based models were able to provide valid estimates of the presence of CSPH and the risk of liver-related events in patients with cACLD [44,45].

A three-parameter model (3P—including platelet count, serum bilirubin, and the international normalized ratio) and a five-parameter model (5P—including platelet count, serum bilirubin, cholinesterase, gamma-glutamyltransferase, and activated partial thromboplastin time) were established. Both models showed accurate diagnostic performance of CSPH, comparable to that of LSM in a merged patient cohort consisting of the internal training data and external validation data sets, with AUCs of 0.775 (3P model) and 0.789 (5P model), compared with to 0.799 for LSM. However, there were high differences reported in the different external validation cohort, indicating that further validation might be required before these models can be adopted in routine clinical practice [45].

### 4.6. Implications for Endoscopic Screening

Given the high diagnostic accuracy and prognostic performance of these non-invasive tools, the necessity of routine endoscopic screening for esophageal varices has become a matter of discussion. Current guidelines suggest that a routine screening endoscopy for the early detection of gastro-esophageal varices as a surrogate marker of CSPH is no longer required, when CSPH can be assumed with high certainty based on non-invasive tests [3].

However, if non-invasive tests are unavailable or yield inconclusive results, endoscopic screening remains justified.

Within the diagnostic ‘grey zone’, endoscopy can reduce the proportion of unclassified patients down to 22% and improve risk stratification [37].

Endoscopy is additionally indicated in patients experiencing first-time decompensation and in patients with intolerance or contraindication to NSBBs, who are at high risk of developing high-risk varices.

Overall, patients with a LSM < 20 kPa and a platelet count > 150 × 10^9^/L, or with a SSM < 40 kPa have a low probability of developing high-risk varices, and endoscopy can therefore be omitted [3,46].

## 5. Novel Techniques for the Assessment of Portal Hypertension

### 5.1. Endoscopic Ultrasound-Guided Portosystemic Pressure Gradient Measurement

Recently developed non-invasive tests require minimal technical resources, are broadly available, cost-effective, easily repeatable and allow a quick risk stratification in patients with advanced chronic liver disease. However, even after the sequential application of different NITs, a relevant proportion of patients remain unclassified in a diagnostic ‘grey zone’ and may therefore still require invasive hemodynamic assessment by HVPG measurement to estimate their risk of hepatic decompensation.

Although HVPG measurement remains the diagnostic gold standard, it has important limitations. Its implementation requires considerable technical expertise and is therefore largely restricted to specialized liver centers. In addition, HVPG primarily reflects sinusoidal portal hypertension and may fail to detect prehepatic or presinusoidal portal hypertension, particularly in the setting of non-cirrhotic portal hypertension and MASLD [22,47,48,49].

Endoscopic ultrasound-guided portal pressure gradient measurement (EUS-PPG) is an emerging technique with the potential to overcome some of these limitations. The method was first introduced by Lai et al. in an animal model in 2004 and later was successfully established in humans [50,51,52].

EUS-PPG is performed by EUS-guided transgastric, transhepatic puncture of the hepatic vein followed by the portal vein, allowing calculation of the pressure gradient between these two vascular compartments. By directly assessing portal venous pressure, EUS-PPG allows for the detection of prehepatic and presinusoidal components of portal hypertension, therefore addressing one of the main limitations of routine HVPG-measurement [48,49]. In a prospective study including 26 patients with porto-sinusoidal vascular disorder (PSVD) and suspected presinusoidal portal hypertension, EUS-PPG measurements were significantly higher than HVPG values, suggesting that EUS-PPG may provide clinically relevant additional information in this setting [47].

Several smaller prospective studies have demonstrated that EUS-PPG correlates closely with HVPG measurements, as well as with clinical features of CSPH, including the presence of esophageal varices, portal hypertensive gastropathy and fibrosis stage [52,53,54,55,56].

Given the widespread availability of EUS in routine clinical practice, EUS-PPG may improve access to portal venous pressure assessment. The method may be particularly attractive in patients requiring concomitant liver biopsy and endoscopic procedures or in those with suspected presinusoidal portal hypertension. However, it remains uncertain whether established HVPG thresholds can be directly transferred to EUS-PPG, and further standardization of EUS-PPG protocols is required to ensure comparability across studies and clinical settings [48,49,53].

### 5.2. Beyond Conventional CT: Radiomics-Based Assessment of Portal Hypertension

The diagnosis of CSPH still primarily relies on the use of NITs and, where available, HVPG measurement [3,6]. In patients with cACLD, however, the presence of ascites, portosystemic collaterals or hepatofugal flow on imaging is sufficient to establish the diagnosis of CSPH [6]. Since the detection of these features on routine imaging may be subtle and observer-dependent, computational imaging approaches are increasingly being explored.

Deep learning and radiomics models applied to routine CT and MRI have shown promising accuracy for the detection of CSPH [57,58].

In this context, de Margerie-Mellon et al. recently demonstrated that combining different imaging modalities, specifically MR liver and spleen elastography with a specific analysis of morphological features such as liver surface nodularity, further improved the prediction of CSPH, with AUC values ranging from 0.80 to 0.89 [59]. Moving beyond classification alone, Sin and coworkers developed a CT-based radiomics model that derived a non-invasive HVPG estimate (‘radio-HVPG’) from contrast-enhanced routine CT scans. This model showed good discriminatory ability for CSPH and performed similarly to invasive-HVPG in predicting liver-related events [60].

Collectively, these data support a growing role for AI-driven imaging biomarkers as scalable and potentially observer-independent tools for the diagnosis and risk stratification of portal hypertension, especially in centers where transient elastography or invasive HVPG measurements are not readily available [57].

## 6. Clinical Management of Portal Hypertension

### 6.1. Pharmacological Treatment Options

NSBBs have been the standard therapy for portal hypertension in patients with liver cirrhosis for more than four decades and remain the only substance class recommended for long-term treatment. The most extensively studied substances include propranolol and carvedilol.

Traditional NSBBs such as propranolol lower portal pressure by reducing portal venous inflow via blockade of sympathetic β1 and β2 adrenergic receptors, the former leading to a reduction in heart rate and cardiac output, the latter to splanchnic vasoconstriction.

Carvedilol, a third-generation NSBB, has a stronger non-selective β-receptor blocking activity (four times higher than propranolol) and additionally blocks the α1-adrenergic system, thereby additionally reducing intrahepatic vascular resistance [61].

Compared with propranolol, carvedilol achieved a greater reduction in HVPG and was associated with a lower rate of hemodynamic non-responders, defined as failure to achieve either an HVPG decrease of more than 20% from baseline or a reduction to an absolute value below 12 mmHg [62,63]. Moreover, carvedilol was able to induce a hemodynamic response in patients who had responded inadequately to propranolol [61,64]. Given its superior portal pressure lowering effect and higher response rate, carvedilol is increasingly regarded as the NSBB of choice [3,61].

Beyond their portal pressure-lowering capacities, NSBBs appear to exert additional beneficial systemic effects. NSBBs have demonstrated anti-inflammatory properties, positively influencing inflammatory biomarkers (e.g., C-reactive protein, interleukin-6, leukocyte count) [14,65]. These anti-inflammatory effects in turn have a positive effect on portal hypertension and can reduce the rate of complications [65]. Furthermore, carvedilol exerts beneficial metabolic effects, and it has been shown that carvedilol may improve glucose tolerance and insulin sensitivity [61,66].

However, the underlying molecular biological mechanisms still need further investigation.

### 6.2. Interventional Treatment Options

Transjugular intrahepatic portosystemic shunt (TIPS) placement is a minimally invasive, image-guided endovascular procedure that creates a shunt between the portal and systemic venous circulation by placing a stent-graft typically between a hepatic vein and the portal vein, thereby reducing HVPG [67].

Beyond its established role in the context of variceal bleeding, either in cases of failure to control bleeding or as secondary prophylaxis in selected patients to prevent rebleeding, TIPS is an effective therapeutic option for other complications of CSPH including refractory ascites or hepatic hydrothorax [68].

Current guidelines support TIPS placement in patients with refractory ascites, defined as ascites resistant to intensive diuretic therapy (spironolactone 400 mg/day and furosemide 160 mg/day) combined with sodium restriction, or intractable ascites, which refers to the occurrence of complications related to diuretic therapy, including hepatic encephalopathy, renal failure or electrolyte imbalance. In addition, TIPS creation is recommended for patients with recurrent ascites, defined by the need for at least three large-volume paracenteses within a 12-month period. In the context of refractory or recurrent ascites, TIPS placement has been associated with improved renal function and haemodynamic parameters, as well as superior ascites control compared with large volume paracenteses [67,68]. Additional indications include refractory hepatic hydrothorax, defined by the need for repeated thoracenteses despite optimized medical therapy, as well as selected patients with Budd–Chiari syndrome or portal vein thrombosis [67,68,69,70].

New approaches also highlight the fact that the placement of a TIPS can be used for the pre-operative conditioning. In selected patients with liver cirrhosis and clinically significant portal hypertension who require major abdominal surgery, a pre-operative TIPS may be considered to reduce perioperative complications. This approach is particularly relevant in patients with refractory ascites, large varices, or evidence of severe portal hypertension who are at high risk for bleeding and postoperative decompensation. By decompressing the portal venous system, pre-operative TIPS can reduce portal pressure, improve ascites control, decrease the risk of perioperative variceal or surgical bleeding, and potentially improve wound healing and postoperative recovery. In carefully selected patients with preserved liver function, pre-operative TIPS may therefore contribute to improved surgical outcomes and lower rates of hepatic decompensation after surgery [71,72].

However, careful patient selection remains essential, as TIPS placement is associated with relevant risks. Absolute contraindications include sepsis or uncontrolled systemic infection, severe cardiac dysfunction, untreated severe valvular heart disease, moderate to severe pulmonary arterial hypertension (mean pulmonary artery pressure > 45 mmHg), severe uncontrolled hepatic encephalopathy, unrelieved biliary obstruction and hepatic lesions (e.g., liver tumors, cysts) precluding TIPS placement [68]. Consequently, a multidisciplinary evaluation is required to balance the potential benefits of TIPS placement against the risks of hepatic decompensation and post-TIPS complications. However, if a TIPS procedure is successful, the patient’s prognosis improves significantly.

### 6.3. Prevention of First Decompensation

The primary therapeutic objective in patients with CSPH is to prevent the first decompensation, as this event is associated with a significant worsening of prognosis. While NSBBs were previously primarily used for secondary prophylaxis after portal hypertensive hemorrhage, there has been a paradigm shift in recent years with a new focus on preventing initial hepatic decompensation through NSBB treatment [2].

The PREDESCI trial showed for the first time that NSBB therapy (propranolol or carvedilol) significantly reduced the risk of first-time hepatic decompensation, especially ascites, in patients with CSPH and compensated cirrhosis [73]. A meta-analysis by Villanueva et al. confirmed that long-term therapy with carvedilol decreased the incidence of decompensation and improved survival in patients with compensated cirrhosis and CSPH [73].

Most recently, Fortea et al. demonstrated in a large multicenter retrospective study that carvedilol was superior to other NSBBs in preventing hepatic decompensation and reducing mortality. Notably, this effect could also be observed in patients with decompensated cirrhosis, in whom long-term therapy with carvedilol was associated with a reduced risk for further decompensation (e.g., refractory ascites, variceal bleeding, etc.) and overall mortality [74].

As a result, the current Baveno VII consensus conference recommend that NSBB therapy, preferably carvedilol, should be considered for all patients with CSPH to prevent hepatic decompensation (Figure 2) [3].

### 6.4. Decompensated Liver Cirrhosis

In patients with cirrhosis, who experienced a first event of decompensation (e.g., ascites), an esophagogastroduodenoscopy (EGD) should still be performed to screen for gastro-esophageal varices, according to the Baveno VII consensus recommendations. If high-risk varices (varices > 5 mm, evidence of red color signs, Child–Pugh class C) are identified, either NSBB therapy or endoscopic variceal ligation (EVL) can be initiated for the primary prevention of esophageal variceal bleeding and other decompensating events, whereby NSBB therapy should be preferred in the absence of contraindications.

If low-risk varices are present (varices < 5 mm, no evidence of red color signs, no Child–Pugh class C), NSBB therapy can be used preferentially to prevent bleeding. This prioritization of NSBB therapy reflects the observation that EVL can prevent the occurrence of esophageal variceal bleeding, while it has no overall systemic effects on CSPH or the occurrence of other decompensation events [3].

Consequently, NSBBs are now considered the preferred substance for primary prophylaxis of portal hypertensive related bleeding in patients with decompensated cirrhosis as they not only reduce the risk of variceal bleeding but also lower the risk of further decompensation and mortality [74,75].

Patients with advanced cirrhosis (Child Pugh B and C) and high-risk varices represent a special risk group, for whom a combined procedure can be considered [76]. In this distinctive risk group, the recent CAVARLY randomized controlled trial demonstrated that a combination therapy of carvedilol and EVL significantly reduced the incidence of first bleeding episodes at one year when compared to carvedilol alone (69.3% vs. 62.9%). Combination therapy also showed a survival benefit, with notably lower overall and bleeding-related mortality rates compared to either monotherapies monotherapy, suggesting its clinical value in selected high-risk patients [76].

### 6.5. Acute Variceal Hemorrhage

If variceal gastrointestinal bleeding is clinically suspected or acute variceal hemorrhage is detected, immediate drug therapy is indicated to lower portal pressure. In this setting, the administration of NSBBs has no clinical relevance, and portal pressure-lowering therapy should be achieved through the administration of other substances.

Terlipressin is the most widely used first-line splanchnic vasoconstrictor (2 mg bolus then 1 mg/6 h for 3–5 days) for acute variceal bleeding in clinical practice [77]. Alternative treatment options include the administration of somatostatin (250 µg as a bolus, then 250 µg/h over 3–5 days) or the administration of octreotide (50 µg as a bolus, then 25 µg/h i.v. for 5 d) [78].

These substances lead to an acute reduction in portal vein pressure via direct splanchnic vasoconstriction; however, they are not suitable for the long-term management of portal hypertension. Recent studies suggest that continuous terlipressin infusion (4 mg/24 h for 5 d) may be superior to intermittent bolus administration, demonstrating better efficacy in terms of HVPG response and a lower rate of adverse events [79]. Nevertheless, the optimal duration of vasoactive drug administration remains a subject of debate. Recent studies indicate that, following successful band ligation, a shorter duration (48–72 h) of vasoactive drug administration does not increase rebleeding rates and may be appropriate in selected patients [80,81].

If the administration of a vasopressor is necessary due to hemodynamic instability, norepinephrine should be preferred while the administration of terlipressin, somatostatin or octreotide should be suspended.

### 6.6. Secondary Prophylaxis of Variceal Hemorrhage

Following an episode of esophageal variceal bleeding, a combined therapy of NSBB and EVL should be initiated for secondary prevention of recurrent hemorrhage [3,78,82].

In patients at high-risk of recurrent hemorrhage (Child–Pugh class B (>7 points) with signs of active bleeding despite vasoactive therapy at the time of the initial endoscopy or Child–Pugh class C (<14 points)), the early placement of a pre-emptive TIPS [83] should be evaluated within 72 h (ideally < 24 h).

The placement of a pre-emptive TIPS significantly reduced the risk of rebleeding or death in selected patients, when compared to standard secondary prophylaxis [84]. Importantly, in patients fulfilling criteria for pre-emptive TIPS, the presence of acute-on-chronic liver failure (ACLF), hepatic encephalopathy or hyperbilirubinemia is not a contraindication. After TIPS placement, further treatment with NSBBs or EVL is not required [3,83,85].

In patients without an indication for TIPS, the optimal choice of NSBBs (e.g., propranolol or carvedilol) in combination with EVL for secondary prophylaxis remains unclear, as only limited comparative data are available [3,86].

In a recent retrospective analysis by Jachs et al., investigating the use of carvedilol or propranolol in the secondary prophylaxis of variceal hemorrhage, carvedilol was associated with a lower re-bleeding rate, a higher HVPG reduction (−20% vs. −11%) and a lower liver-associated mortality [87].

Although current evidence suggests a potential superiority of carvedilol over traditional NSBBs in secondary prophylaxis, further prospective studies are needed to confirm the efficacy and benefits of carvedilol.

### 6.7. Practical Information on the Use of NSBBs

NSBBs should always be dosed gradually. The initial dosages are 6.25 mg (single dose) per day for carvedilol and 20–40 mg per day for propranolol (divided into a morning and evening dose). If well tolerated, carvedilol may be increased to the target dose of 12.5 mg/day after 3 days, whereas propranolol should be up-titrated every 3 days to a target dose of 80–160 mg/day (divided into a morning and evening dose).

Higher doses of carvedilol (>12.5 mg/day) should only be administered if there is an additional indication such as the presence of arterial hypertension.

Dose escalation should be halted if systolic blood pressure falls below 90 mmHg, mean arterial pressure below 65 mmHg, or resting heart rate below 55/min (for propranolol). Previous analyses have shown a lower rate of side effects with carvedilol compared to traditional NSBBs such as propranolol.

However, it should always be considered that only 30–60% of patients show an adequate response to NSBB treatment, while in the remaining patients, treatment has to be discontinued due to side effects or inadequate reduction in HVPG [64].

Absolute contraindications to NSBB therapy include uncontrolled asthma, severe bradycardia (<50 bpm), a 2nd or 3rd degree atrio-ventricular block, sick sinus syndrome, cardiogenic shock, critical limb ischemia or a known hypersensitivity to NSBBs. In addition, the administration of NSBBs should be critically evaluated, and NSBB therapy should be temporarily discontinued or dose-adjusted in the presence of hypotension (systolic blood pressure < 90 mmHg or mean arterial pressure < 65 mmHg), sepsis, refractory ascites, spontaneous bacterial peritonitis, acute renal failure with a creatinine value > 1.5 mg/dL or hepato-renal syndrome [6,88].

Common adverse effects of NSBBs that should be regularly assessed include dizziness, fatigue and erectile dysfunction.

Notably, recent studies suggest that the HVPG response to NSBB therapy may be limited in patients with compensated cirrhosis who also have type 2 diabetes mellitus and an increased BMI. This should be taken into consideration when evaluating apparent treatment failures in this subgroup [89].

### 6.8. Evaluation of the Treatment Response

Due to the increasing use of NSBBs, systemic evaluation of the treatment response and the identification of ‘non-responders’ have become clinically highly relevant.

Since invasive HVPG measurement to assess the treatment effect of NSBBs is complex and only available at specialized centers, non-invasive methods for evaluating treatment response are attractive alternatives.

One promising option here is the measurement of spleen stiffness [90]. In a prospective study, Kim et al. demonstrated that dynamic changes in spleen stiffness, measured by using Acoustic Radiation Force Impulse (ARFI), could reflect the hemodynamic response to carvedilol, enabling the discrimination between responders and non-responders [91]. Marasco et al. confirmed this observation, by demonstrating that a reduction in spleen stiffness of more than 10% measured by transient elastography in response to primary prophylactic carvedilol therapy successfully predicted a change in HVPG after three months of NSBB therapy (100% sensitivity, 60% specificity) in patients with high-risk esophageal varices [92]. In contrast, changes in spleen stiffness assessed by magnetic resonance elastography did not correlate reliably with invasive HVPG measurements following NSBB therapy [93]. These discrepant results indicate that further prospective studies are needed to determine which diagnostic methods of spleen stiffness measurement are suitable for adequately evaluating treatment response [93].

Further promising non-invasive methods for evaluating treatment response with NSBBs are metabolome and lipidome analyses, although these methods do require further scientific investigation and have not been used in clinical practice yet [94,95].

In summary, the increasing clinical use of NSBBs highlights the need for intensified research efforts to establish reliable non-invasive biomarkers for treatment monitoring and for the early identification of non-responders.

### 6.9. Recompensation

Hepatic recompensation has emerged as an important concept in the clinical course of advanced chronic liver disease, reflecting the potential for partial regression of structural and functional liver impairment following effective etiological therapy [96,97].

According to the Baveno VII consensus, recompensation is defined by fulfillment of all of the following criteria: (i) elimination or suppression of the underlying cause of cirrhosis; (ii) resolution of prior events of decompensation, including ascites and hepatic encephalopathy without ongoing specific therapy, as well as the absence of recurrent variceal bleeding for at least 12 months; and (iii) a sustained improvement in liver function parameters [3].

However, while recompensation has been associated with improved survival, the risk of developing HCC remains elevated compared with patients who have never experienced decompensation. Therefore, surveillance for HCC should be continued. Furthermore, recompensation does not necessarily coincide with resolution of CSPH, which may persist despite clinical and biochemical improvement [96,98,99]. The course of portal hypertension and its optimal management after recompensation remain subjects of ongoing research. In this context, NITs may provide valuable information for the assessment of CSPH after recompensation has been achieved. Recent data in patients with recompensated alcohol-related cirrhosis suggest that LSM, SSM, and vWF-Ag correlate with HVPG dynamics following recompensation and may assist in identifying persistent CSPH. In particular, elevated LSM values (≥25 kPa) continue to reliably indicate ongoing CSPH, whereas lower LSM values (≤15 kPa) were less accurate for ruling out CSPH and are not improved by combination with platelet count. Notably, platelet count demonstrated poor correlation with HVPG and limited diagnostic performance for assessing CSPH resolution after recompensation [100].

Overall, these findings support a potential role for selected NITs in the longitudinal evaluation of CSPH following recompensation. Future studies should focus on validating NIT-based algorithms in recompensated cirrhosis and refining cutoff values, particularly for CSPH resolution, to better guide clinical decisions regarding portal hypertension-directed therapies following recompensation.

## 7. Outlook—Emerging Drugs for the Treatment of Portal Hypertension

In addition to NSBBs, further substances show promising effects on the reduction in portal hypertension:

### 7.1. Statins

Beyond their lipid-lowering properties, statins exert multiple pleiotropic effects through various signaling pathways, including antifibrotic, anti-inflammatory, antioxidant, and antiproliferative actions, as well as enhanced NO bioavailability [101,102].

In addition, statins reduce intrahepatic vascular resistance. Abraldes et al. demonstrated that statin therapy lowered HVPG and improved survival following variceal hemorrhage [103]. In a recent study investigating alternative treatment regimens in patients with suboptimal response to β-blockers (HVPG decrease < 20%), Alvarado-Tapias et al. showed, that a combination therapy of carvedilol and simvastatin significantly enhanced the portal pressure reduction achieved with carvedilol monotherapy (2.97 vs. 2.05 mmHg). This regimen also improved endothelial dysfunction, and reduced proinflammatory cytokines [104].

### 7.2. Anticoagulants

The occlusion of small hepatic vessels due to fibrotic remodeling processes, endothelial damage and microthrombi is associated with the progression of cirrhosis and an increase in intrahepatic vascular resistance. Enoxaparin, a low-molecular-weight heparin, and rivaroxaban, a direct oral anticoagulant, reduced intrahepatic vascular resistance in preclinical studies in cirrhotic rat models, which was associated with a significant reduction in liver fibrosis, activation of hepatic stellate cells and lower rates of intrahepatic microthrombosis [105,106]. In patients with cirrhosis who were on a waiting list for a liver transplant, one year of treatment with enoxaparin also reduced the occurrence of hepatic decompensation. Based on these preclinical and clinical observations, it is assumed that anticoagulant substances have favorable effects on the severity of portal hypertension by reducing intrahepatic vascular resistance [107].

### 7.3. FXR-Receptor-Agonists

The farnesoid X receptor (FXR) is a nuclear transcription factor that is expressed in the liver, intestine and kidney and is activated by bile acids. Preclinical studies have shown that FXR activation by FXR agonists was associated with a reduction in portal hypertension by reducing liver fibrosis, vascular remodeling and sinusoidal dysfunction [108]. In a small clinical study, the administration of the FXR agonist obeticholic acid over 7 days in patients with alcohol-associated liver disease resulted in a reduction in HVPG [109]. However, it has to be mentioned that the use of obeticholic acid in patients with advanced cirrhosis can lead to decompensation and even death, so that the benefits of this drug must be critically scrutinized [110]. Consequently, the extent to which newer FXR agonists (e.g., Cilofexor, Tropifexor) can influence the severity of portal hypertension needs further investigation.

### 7.4. PPAR-Agonists

Peroxisome proliferator-activated receptors (PPARs) form a family of nuclear transcription factors that, among other functions, can mitigate inflammation, fibrosis, and insulin resistance in liver disease. In preclinical studies, the pan-PPAR agonist Lanifibranor has demonstrated efficacy in improving portal hypertension by reducing liver fibrosis and hepatic vascular tone. Nevertheless, additional studies are required to translate these findings into clinical practice [9,111,112].

Further substances that have shown antifibrotic and portal pressure-reducing effects in pre-clinical and initial smaller clinical studies include Glucagon-like peptide-1 (GLP-1) agonists (liraglutide, semaglutide), endothelin receptor antagonists, phosphodiesterase inhibitors and the galectin-3 inhibitor belapectin. These drugs were able to reduce oxidative stress, inflammation and activation of hepatic stellate cells.

However, further prospective studies which show their effective benefits are still pending [113].

## 8. Conclusions

Cirrhotic patients with CSPH represent a particularly vulnerable risk group, as the occurrence of a decompensating event is associated with a significant worsening in prognosis.

While invasive HVPG measurement remains the gold standard for assessing portal hypertension, advances in non-invasive diagnostics nowadays allow an accurate identification and risk stratification of patients with CSPH through the assessment of liver stiffness, platelet count, and spleen stiffness.

Among NSBBs for treating CSPH, carvedilol induces a greater reduction in portal vein pressure than traditional NSBBs (e.g., propranolol) due to its additional α1-adrenergic antagonistic properties and should therefore be used preferentially.

The incorporation of non-invasive diagnostics into standard clinical practice, in addition to effective pharmacological treatment, presents a broadly accessible, reliable, and cost-effective approach to optimize risk stratification and improve outcomes in patients with liver cirrhosis.

## 9. Take-Home Messages

- In patients with cirrhosis, CSPH represents a key driver of hepatic decompensation, which is associated with a significant worsening in prognosis.
- Although HVPG measurement remains the diagnostic gold standard, NITs—including LSM, SSM, platelet count or vWF-Ag—enable accurate identification and risk stratification of patients with CSPH.
- Combined NIT-based algorithms (e.g., Baveno criteria, ANTICIPATE±NASH, NICER model, VITRO Score) substantially improve diagnostic accuracy and reduce the proportion of patients remaining in a diagnostic ‘grey zone’ for the diagnosis of CSPH.
- NSBBs remain the cornerstone of medical therapy in CSPH, with carvedilol being the preferred agent due to its superior portal pressure-lowering effect.
- Emerging concepts such as hepatic recompensation highlight the potential dynamic clinical course of cirrhosis; however, CSPH may persist and the risk of HCC remains elevated, necessitating continued monitoring and surveillance.
- Overall, the incorporation of non-invasive diagnostics into standard clinical practice for early detection of CSPH, combined with effective pharmacological treatment, is essential to improve clinical outcomes in patients with portal hypertension.

## Figures and Tables

**Table 1:** Comparison of non-invasive tests.

Non-Invasive Tests	Parameters	Proposed Cut-Offs	Strengths	Limitations
Liver stiffness by transient elastography	LSM [kPa]		Short learning curve, can be performed by trained technicians	High risk of confounders
			Easily repeatable	Does not capture presinusoidal/prehepatic components of PH
				Influenced by recent food intake
Baveno VII criteria	LSM [kPa]	Rule-out CSPH:	Extensively validated and broadly applicable in cACLD	Large ‘grey zone’
		LSM ≤ 15 kPa + PLT > 150 × 10^9^/L		
	PLT [×10^9^/L]	Rule-in CSPH:	High PPV and NPV for ruling-in/-out CSPH	Not validated for obese patients with MASLD (BMI > 30 kg/m^2^)
		LSM ≥ 25 kPa		
ANTICIPATE	LSM [kPa]	Presume CSPH:	Easy to assess	Not validated for obese patients with MASLD (BMI > 30 kg/m^2^)
	PLT [×10^9^/L]	LSM 15–20 kPa+ PLT < 110 × 10^9^/L	Provide a continuous risk prediction of the probability of having CSPH	
		LSM 20–25 kPa + PLT < 150 × 10^9^/L		
ANTICIPATE-NASH	LSM [kPa]	CSPH risk threshold ≥ 60%	Validated for obese patients with MASLD (BMI > 30 kg/m^2^)	Primarily developed for a specific subgroup
	PLT [×10^9^/L]			
	BMI [kg/m^2^]	(≥75% improves rule-in performance)	(Improves PPV, compared with LSM ≥ 25 kPa alone)	
Spleen stiffness by transient elastography	SSM [kPa]	Dual cut-off:	Reflects hemodynamic consequences of PH more closely than LSM	Results are impacted by recent food intake
		Rule-out CSPH: SSM < 21 kPa		Higher technical failure rate, especially with older 50 Hz-probes
		Rule-in CSPH: SSM > 50 kPa		
		Single cut-off (in combination with LSM ± PLT):	Dual cut-off can rule-in/rule-out the presence of CSPH with high accuracy	Influenced by recent food intake
		Rule-in: SSM > 40 kPa	Reduces the ‘grey-zone’, when added to Baveno VII criteria as Single-cut-off.	Less widely available
		Rule-out: SSM ≤ 40 kPa		
NICER Model	LSM [kPa]		Continuous risk prediction model	No single universally used bedside cut-off
	SSM [kPa]			
	PLT [×10^9^/L]		Highest discriminative ability for CSPH among current NIT-based models	
	BMI [kg/m^2^]		Validated across all ACLD etiologies (including obese MASLD)	
VITRO-Score	vWF-Ag [%]	Rule-out: ≤1.5	Broadly available laboratory-based score	Acute phase protein, subject to diurnal variations, influenced by statin-treatment
	PLT [×10^9^/L]	Rule-in: ≥2.5	Useful adjunct after Baveno VII criteria, can reduce the ‘grey-zone’.	Assay-related variability may limit generazibility

Abbreviations: BMI, Body mass index; cACLD, compensated advanced chronic liver disease; CSPH, clinically significant portal hypertension; kPa, Kilopascal; LSM, Liver stiffness measurement; MASLD Metabolic dysfunction-associated steatotic liver disease; NPV, negative predictive value; PLT, platelet count; PPV, positive predictive value; SSM, Spleen stiffness measurement; VITRO, Von Willebrand Factor Antigen/Thrombocyte Ratio; vWF-Ag: von Willebrand factor antigen.

## Data Availability

All data generated or analyzed during this study are included in the manuscript. For additional information or access to raw data, please contact the corresponding authors.

## Abbreviations

The following abbreviations are used in this manuscript:

ACLF	Acute-on-chronic liver failure
ARFI	Acoustic Radiation Force Impulse
AUROC	Area under the receiver operating characteristic curve
BMI	Body mass index
cACLD	Compensated advanced chronic liver disease
CO	Carbon monoxide
CSPH	Clinically significant portal hypertension
DAMPs	Damage-associated molecular patterns
EGD	Esophagogastroduodenoscopy
EVL	Endoscopic variceal ligation
FHVP	Free hepatic venous pressure
FXR	Farnesoid X receptor
GLP-1	Glucagon-like peptide-1
HM/HMs	Hepatic macrophages
HSC	Hepatic stellate cells
HVPG	Hepatic venous pressure gradient
H_2_O	Water
IHVR	Intrahepatic vascular resistance
kPa	Kilopascal
LSM	Liver stiffness measurement
LSECs	Liver sinusoidal endothelial cells
MAP	Mean arterial pressure
MASLD	Metabolic dysfunction-associated steatotic liver disease
MAMPs	Microbiome-associated molecular patterns
NASH	Non-alcoholic statohepatitis
NITs	Non-invasive tests
NICER	Non-invasive CSPH estimated risk (model)
NO	Nitric oxide
NSBBs	Non-selective beta-blockers
PAMPs	Pathogen-associated molecular patterns
PLT	Platelet count
PGI_2_	Prostacyclin (Prostaglandin I_2_)
PPAR(s)	Peroxisome proliferator-activated receptor(s)
RAAS	Renin–angiotensin–aldosterone system
ROS	Reactive oxygen species
SNS	Sympathetic nervous system
SSM	Spleen stiffness measurement
TIPS	Transjugular intrahepatic portosystemic shunt
TE	Transient elastography
TLR	Toll-like receptor
VITRO	Von Willebrand Factor Antigen/Thrombocyte Ratio
vWF-Ag	Von Willebrand factor antigen
WHVP	Wedged hepatic venous pressure
